# Account of the Use of an Instrument for Cutting the Cornea, in the Operation of Extracting a Cataract

**Published:** 1799-06

**Authors:** A. Carlisle

**Affiliations:** Surgeon to the Westminster Hospital


### *Account of the Use of an Instrument for cutting the Cornea, in the Operation of extracting a Cataract.*

*By* A. CARLISLE, *Surgeon to the Westminster Hospital.*

A Few years ago, it fell in my way to see an instrument which had been used with success at Paris, and in other parts of the Continent of Europe, for making the incision of the cornea, in the operation of extraction. The great difficulty of finding the globe of the eye, and the consequent tediousness, as well as incertitude of performing the incision, induced me to try the instrument to be described.

Having previously decided in my own judgment, that depression is a less efficacious operation than extraction, provided the latter be performed with due attention and dexterity, and having found that nearly the whole of the success turned upon the incision of the cornea, the following case presently occurred:—A blind woman, having had one eye spoiled by an attempt at depression, and the other useless from a complete cataract, was pitched upon for the operation. The subject was a very indifferent one, being old, and afflicted with chronic inflammation of the eye-lids. The globe of the eye, however, during the operation, was tranquil; the incision was made satisfactorily, and, without any other assistance, the opaque crystalline lens jumped out.

out. An undue degree of preſſure occaſioned the flow of a ſmall quantity of the vitreous humour, nevertheleſs the patient recovered her perfect ſight. Mr. Lynn, ſenior ſurgeon of the Weſtminſter Hoſpital, has alſo employed the ſame inſtrument with perfect ſucceſs as to the inciſion; but as the caſes are both now under treatment, the reſult cannot be aſcertained.

The form and particular mechaniſm of this inſtrument is not eaſily deſcribed in words; and an expenſive plate would only gratify curioſity, without enabling the inſtrument-maker to form one with that accuracy which this nice operation requires. Mr. Stodart, an eminent ſurgeon's inſtrument maker, in the Strand, has paid particular attention to this inſtrument, has ſeen it uſed, and made himſelf acquainted with all its needful qualifications; he has it for ſale. It conſiſts of a plate of braſs, with a loop, or ring, of the ſize of the tranſparent cornea, fixed at a right angle with one end of the plate. The eye-lids being held open, the cornea is made to project through this ring, ſo as to expoſe nearly the whole of its tranſparent part beyond its inner edge. A cutting blade of ſteel is made to ſlide cloſe againſt the inner ſurface of this braſs loop, which is acted upon by a ſtrong ſteel ſpring. When cocked, the blade is carried beyond the right edge of the ring; and when let off by a trigger, it paſſes through the lower half of the cornea, cutting a flap of a ſemicircular form at its lower edge. The other part of the operation is then to be conducted as uſual. An adjuſtable ſcrew alters the diſtance of the cutting blade from the ring through which the cornea protrudes, ſo as in ſome meaſure to adapt the inſtrument to eyes of different diameters. The ring itſelf is alſo made removeable, ſo that one of the ſize required may be choſen.

If there is any objection to this mode of dividing the cornea, it reſts with the circumſtance of the ſudden collapſe of the globe of the eye, endangering the protruſion of the vitreous humour, before the muſcles of the globe are ſufficiently aware of the reſiſtance taken from that body, or before they recover from the ſhock which this inciſion communicates to the whole organ. Still, however, taking into conſideration the difficulty, and even ſometimes total impoſſibility of making a good inciſion with the hand, I ſhould conceive that the uſe of this inſtrument may be attended with beneficial conſequences in this country,

*Account*

