# Errata

**Published:** 1799-07

**Authors:** 


No. IV. p. 332, l. 13, from the bottom for "finding" read, "*fixing* the globe of the eye.

